# COVID-19 Outbreak Can Change the Job Burnout in Health Care Professionals

**DOI:** 10.3389/fpsyt.2020.563781

**Published:** 2020-12-08

**Authors:** Xinghuang Liu, Jie Chen, Dongke Wang, Xin Li, Erchuan Wang, Yu Jin, Yanling Ma, Cheng Yu, Chang Luo, Lei Zhang, Chuang Liu, Yangshiyu Zhou, Ling Yang, Jun Song, Tao Bai, Xiaohua Hou

**Affiliations:** ^1^Division of Gastroenterology, Union Hospital, Tongji Medical College, Huazhong University of Science and Technology, Wuhan, China; ^2^Department of Paediatrics, Union Hospital, Tongji Medical College, Huazhong University of Science and Technology, Wuhan, China; ^3^Department of Respiratory and Critical Care Medicine, Union Hospital, Tongji Medical College, Huazhong University of Science and Technology, Wuhan, China; ^4^Ultrasonic Department, Union Hospital, Tongji Medical College, Huazhong University of Science and Technology, Wuhan, China; ^5^Wuhan Estrip Tech Co., Ltd., Wuhan, China

**Keywords:** SARS-CoV-2 (2019-nCoV), coping style, health care professional, COVID-19, job burnout

## Abstract

**Background:** The outbreak of COVID-19 in China was a sudden bio-disaster, which may bring a negative impact on the job burnout of health care professionals (HCPs).

**Objective:** We aim to find out the association factors, especially those closely related to this outbreak, of job burnout in Chinese HCPs.

**Method:** The cross-sectional survey about HCPs' job burnout based on a network platform was conducted in high and low infection regions during the COVID-19 outbreak in China. The demographic characteristics, medical-work-related factors, risk of getting infected due to occupational exposure, and family factors were collected by the self-reported questionnaire. The Chinese version of the Maslach Burnout Inventory (CMBI) and the Trait Coping Style Questionnaire (TCSQ) were employed in this study to evaluate the job burnout and coping style, respectively. Furthermore, statistical analysis was done to find out the associated factors of job burnout.

**Results:** We collected 880 complete questionnaires from doctors and nurses from February 9, 2020 to February 11, 2020. In this study, the positive rates of three dimensions of burnout (emotional exhaustion, depersonalization, and reduced personal accomplishment) and overall burnout were 9.09, 50.57, 56.59, and 73.98%, respectively. After the statistical analysis, we found that several factors can independently affect the dimensions. Working in the high infection region and negative coping styles can affect all three dimensions at once. More night shift quantity and having symptoms could increase emotional exhaustion and depersonalization, while higher work intensity and senior title could increase emotional exhaustion and reduce personal accomplishment, respectively.

**Conclusion:** The rate of moderate and severe burnout had increased due to the outbreak. More attention should be paid to burnout in HCPs, especially those with negative coping. There were some potential ways to reduce burnout, such as reducing their workload and providing better protection from the virus.

## What Is New?

### Key Findings

This study showed the effect of several factors related to work, family, and individual characteristics on job burnout symptoms in Chinese health care professionals.

What this adds to what is known: This study focused on the status of job burnout in the pandemic of COVID-19 in Chinese health care professionals. We conduct this study to add evidence to what impact the public health emergency can bring to job burnout symptoms of health care professionals, which has been rarely studied before.

What is the implication and what should change now: More attentions need to be paid to the phenomenon of job burnout in Chinese health care professionals. Reducing workload, providing more job resources, and carrying out psychological interventions for those with negative coping style may be the potential ways to alleviate job burnout.

## Introduction

At the beginning of 2020, COVID-19 spread across China. The outbreak of COVID-19 was adding to the pressure on Chinese health care professionals (HCPs), already overburdened by a large population and growing health awareness in recent years. A large number of doctors and nurses were on the front line in the fight against this highly contagious virus, facing increased workload, high risk of infection, and the pressure of isolating from family members. Those stressors caused by this public health disaster may arise negative emotions and even job burnout in HCPs.

Burnout was defined as a syndrome of emotional exhaustion (EE), depersonalization (DP), and reduced professional accomplishment (PA) that occurs among various people-oriented professions, including doctors and nurses (1). Emotional exhaustion was a key aspect of the syndrome and refers to the feeling that a person's emotional resources were overextended and depleted. Depersonalization refers to negative, cynical, cold, and impersonal attitudes and feelings toward others. Finally, decreased personal accomplishment refers to a person's decreased sense of competence and a tendency to negatively evaluate oneself, especially in terms of cooperation with others (2).

According to previous studies, job burnout symptoms were common in health system practitioners (3–6). Job-related pressure and personal characteristics contributed to the development of job burnout symptoms together. Besides, moral distress and stress related to physical and mental environments are also thought to contribute to burnout (7). The most direct impacts of job burnout on HCPs were the high rate of turnover and the low efficiency in daily work (5). It meant that patients would receive medical service in poor quality, and the national health system needs to pay extra expenses for the replacement of new physicians, which was not a small cost (8, 9). There was evidence that job burnout was associated with multiple mental disorders, including anxiety, depression, and a decrease in self-esteem (5, 10). The relationship between job burnout and physical symptoms, such as insomnia and headache, has also been reported (6). By contrast, finding and implementing ways to reduce burnout must help both HCPs and patients.

Coping styles were considered as stable strategies that can overcome or tolerate external and internal pressures or stresses (11). Based on Lazarus and Folkman's model, coping was defined as “constantly changing cognitive and behavioral efforts to manage specific external and/or internal demands that are appraised as taxing or exceeding the resources of a person (12).” Some people react to stress actively, while others react passively. Positive coping styles were behavioral or psychological responses (such as using emotional supports and positive reframing) designed to change the nature of the perspective of the stressor (13). Negative coping strategies could cause people to engage in activities (such as drinking) that prevented them from dealing directly with stressful events (14). What is more, personality characteristic of a negative coping style was identified as risks and a positive coping style as protective against some mental disorders, including anxiety, depressive, adjustment, and somatoform disorders (15). Individuals' coping behaviors help explain why they are exposed to the same environment that may cause job burnout in some subjects, but not in others (16). Previous studies had shown that the coping style played the mediator and mediating role in the development of burnout and could be a positive resource against burnout (17, 18).

HCPs must take care of themselves before they could provide care for their COVID-19 patients. Given the current high intensity of work, work exposure risks, different coping strategies, and isolation from family members, we needed to understand the situation of burnout and elucidate the association between job burnout and those factors among Chinese HCPs. Meanwhile, many experts were also calling attention to the problem of burnout among HCPs (19, 20). The good news was that experts have come up with some effective micropractices to prevent burnout since the outbreak (21). However, in this pandemic of COVID-19, the situation of job burnout has not been fully investigated in China. For calling more attention to the job status of HCPs who were fighting the virus, we designed this cross-sectional study to observe the prevalence of job burnout and analyze its risk factors.

## Method

### Design

A cross-sectional survey based on the Internet was conducted from February 9 to February 11, 2020, and was approved by the Ethics Committee of Union Hospital, Tongji Medical College. By self-reported questionnaires, we identified our target respondents (doctors and nurses) and collected the information we need while protecting their privacy.

The e-questionnaire was distributed on the online platform, and before the questionnaire starts, the respondents could see our self-introduction, our research purpose, and the introduction of the questionnaire content. Only after the respondents choose to agree to the survey the content of the questionnaire would be launched; otherwise, the survey would be terminated directly. Respondents could terminate the questionnaire at any time and choose whether to submit it or not after completing the questionnaire. So, respondents' right to know would also be protected, and the questionnaires we received were approved by them. There were no privacy issues, and the questionnaire was anonymous. The questionnaire was written in Chinese, and each question was interpreted to avoid ambiguity. By setting up the network platform, we only allowed one respondent to fill in one questionnaire, and only the completed questionnaire would be collected.

### Questionnaire

The information collected by the questionnaire could be divided into the following six aspects: (a) demographic characteristics; (b) working factors during the outbreak; (c) risk assessment of SARS-CoV-2 infection; (d) family factors; (e) coping style assessment by TCQS; and (f) job burnout assessment by CMBI.

The demographic characteristics included age, sex, occupation (doctor, nurse, or others), and the title of occupation (primary title, senior title, none). Primary title included medic, resident doctor, attending doctor, primary nurse, and primary nurse practitioner; senior title included associate chief physician/associate professor, chief physician/professor, nurse-in-charge, deputy chief nurse, and senior nurse.

Working factors included working hours per week during the COVID-19 outbreak, the number of night shifts per week during the same period, and self-reported work intensity (higher than that before the outbreak or unchanged). Therefore, the change in work intensity measured in this article referred to the change due to the recent COVID-19 pandemic.

Risk assessment of SARS-CoV-2 infection included the common COVID-19 symptoms (fever and respiratory symptoms), whether COVID-19-related tests (CT scan and Viral nucleic acid test) had been conducted, the results, whether working in Hubei province, China, and whether working in the front line (directly contacting with confirmed COVID-19 patients at work). HCPs who reported nucleic acid positive or CT positive would not be included in the study because they would be quarantined and removed from work, as our research focused on people who were fighting the virus in hospitals. During the survey period, more than 31,000 people were diagnosed in Hubei province, compared with a national total of about 42,000 (22). Therefore, we defined Hubei province as the high infection region, and other areas in China were defined as the low infection region.

Family factors referred to whether HCPs were isolated from family members because of their work exposure.

### Assessment of Coping Style

The 20-item Chinese Trait Coping Style Questionnaire (TCSQ) was chosen in this study to assess the coping style (positive coping or negative coping) of respondents. In terms of the two coping styles, the questionnaire set 10 multiple-choice questions for evaluation, and these 10 questions were presented to the interviewees in an interwoven order. Each question was rated on a scale at five levels (one score for definitely no, and five scores for definitely yes) (17, 23, 24). The score of coping tendency was equal to the score of positive coping dimension minus the score of negative coping dimension. A positive score meant positive coping style, while a negative score or zero means negative coping style (10, 17). The TCSQ was proved to be valid and reliable in the Chinese population (25). In this study, the Cronbach's α value for both coping styles was 0.858. As a rule of thumb, this α value meant that the questionnaire had a good internal consistency (26).

### Assessment of Job Burnout

The 15-item Chinese Maslach Burnout Inventory (CMBI) revised by Li et al. (27) was used in this study. The CMBI scale consisted of three dimensions of burnout: emotional exhaustion (EE) (five items), depersonalization (DP) (five items), and reduced personal accomplishment (RPA) (five items). The projects were rated on a seven-point scale, ranging from 1 (never) to 7 (every day). The five items about reducing individual achievement were reversely coded (17). The cutoff scores for the three dimensions (EE, DP, and RPA) were 25, 11, and 16, respectively, according to the evaluation criterion (17, 28). The CMBI scale's good reliability and validity, especially in the Chinese population, were proved by many studies (28–30). In this study, the Cronbach's α value for the whole scale was 0.832. Meanwhile, the α values were 0.936, 0.912, and 0.931 for EE, DP, and RPA, respectively. This α value also indicated that the internal consistency of the scale was good (26).

In this study, according to the positive (higher than the cutoff score of the dimension) number of respondents in three dimensions, we divided job burnout into four levels: no burnout (all the three dimensions are negative); mild burnout (only one of the three dimensions is positive); moderate burnout (arbitrary two of the three dimensions are positive); and severe burnout (all the three dimensions are positive) (31, 32).

### Statistical Analyses

It was just the questionnaire that the doctor or the nurse fills out that would be included in our study. Data of questionnaires were cleaned, coded, and double-entered using EpiData software 3.1. Another software, STATA 14.0 (http://www.stata.com), was used for data analysis. Two-sided *P* < 0.05 was considered statistically significant.

Continuous variables were analyzed employing Student's *t*-test or Wilks' lambda test when required. Categorical variables were compared via the chi-square test or Fisher's exact test as appropriate. Stepwise binary logistic regressions were used to determine the effect of the independent factors on the job burnout in the three dimensions separately.

## Result

All the 880 questionnaires completed by HCPs were collected through the online platform, of which 564 were doctors and 316 were nurses (Table 1). Although some of the HCPs interviewed were tested (CT or nucleic acid), none came back positive, which indicated that there was no confirmed diagnosis among them. Overall, 80 (9.09%) respondents had emotional exhaustion (EE), 445 (50.57%) had depersonalization (DP), and 498 (56.59%) had reduced personal accomplishment (RPA) (Figure 1). The proportions of job burnout were showed mild with 34.77%, moderate with 36.14%, and severe with 3.07%.

**Table 1:** Distributions of dimensions of burnout in categorical items.

**Aspects**	**Items**	***N***	**EE yes/no (%)**	**DP yes/no (%)**	**RPA yes/no (%)**
**Demographic** ** characteristics**	**Age group**		*P* = 0.343	*P* = 0.015^*^	*P* = 0.069
	20–29	198	16/182 (8.08%)	98/100 (49.49%)	128/70 (64.65%)
	30–39	406	43/363 (10.59%)	225/181 (55.42%)	224/182 (55.17%)
	40–49	191	17/174 (8.90%)	90/101 (47.12%)	101/90 (52.88%)
	≥50	85	4/81 (4.94%)	32/53 (37.65%)	45/40 (52.94%)
	**Occupation**		*P* = 0.673	*P* = 0.594	*P* = 0.333
	Doctor	564	53/551 (9.40%)	289/275 (51.24%)	326/238 (57.80%)
	Nurse	316	27/289 (8.54%)	156/160 (49.37%)	172/144 (54.43%)
	**Gender**		*P* = 0.271	*P* = 0.655	*P* = 0.649
	Male	279	21/258 (7.52%)	138/141 (49.46%)	161/118 (57.71%)
	Female	601	59/542 (9.82%)	307/294 (51.08%)	337/264 (56.07%)
	**Title**		*P* = 0.354	*P* = 0.006^**^	*P* = 0.013^*^
	Primary	328	26/302 (7.93%)	146/182 (44.51%)	168/160 (51.22%)
	Senior	552	54/498 (9.78%)	299/253 (54.17%)	330/222 (59.78%)
Working factors	**Working hours^#^**		*P* = 0.008^**^	*P* = 0.621	*P* = 0.378
	Yes (mean ± SD)		52.86 ± 3.07	46.71 ± 1.02	45.72 ± 0.98
	No (mean ± SD)		45.68 ± 0.80	45.94 ± 1.19	47.12 ± 1.26
	**Work intensity**		*P* < 0.001^***^	*P* = 0.967	*P* = 0.658
	Higher	406	62/334 (15.27%)	205/201 (50.49%)	233/173 (57.39%)
	Basically unchanged	474	18/456 (3.80%)	240/234 (50.63%)	265/209 (55.91%)
	**Night shift quantity**		*P* < 0.001^***^	*P* = 0.005^**^	*P* = 0.891
	0–1	475	22/453 (4.63%)	217/258 (45.68%)	266/209 (56.00%)
	2–3	358	51/307 (14.25%)	204/154 (56.98%)	206/152 (57.54%)
	≥4	47	7/40 (14.89%)	24/23 (5.11%)	26/21 (5.53%)
The potential risk of infection	**Symptom**		*P* < 0.001^***^	*P* = 0.008^**^	*P* = 0.738
	Asymptomatic	698	44/654 (6.30%)	337/361 (48.28%)	397/301 (56.88%)
	Symptomatic	182	36/146 (19.78%)	108/74 (59.34%)	101/81 (55.49%)
	**Working place**		*P* < 0.001^***^	*P* = 0.294	*P* = 0.011^*^
	High infection region	395	58/337 (14.68%)	192/203 (48.61%)	205/190 (51.90%)
	Low infection region	485	22/463 (4.54%)	253/232 (52.16%)	293/192 (60.41%)
	**Contact with patients**		*P* < 0.001^***^	*P* = 0.338	*P* = 0.135
	Direct	258	45/213 (17.44%)	124/134 (48.06%)	136/122 (52.71%)
	Indirect	622	35/587 (5.63%)	321/301 (51.61%)	362/260 (58.20%)
Family factor	**Family life**		*P* < 0.001^***^	*P* = 0.919	*P* = 0.822
	Isolation from family	199	36/163 (18.09%)	100/99 (50.25%)	114/85 (57.29%)
	Live with family	681	44/637 (6.46%)	345/336 (50.66%)	384/297 (56.39%)
TCQS	**Coping style**		*P* < 0.001^***^	*P* < 0.001^***^	*P* < 0.001^***^
	Negative coping	276	39/237 (14.13%)	195/81 (70.65%)	182/94 (65.94%)
	Positive coping	604	41/563 (6.79%)	250/354 (41.39%)	316/288 (52.32%)

* p < 0.05;

** *p < 0.01*;

*** *p < 0.001*.

# *Working hours is a continuous variable, so “yes” means that the corresponding dimensions are positive, and “no” means that the corresponding dimensions are negative*.

**Figure 1:**
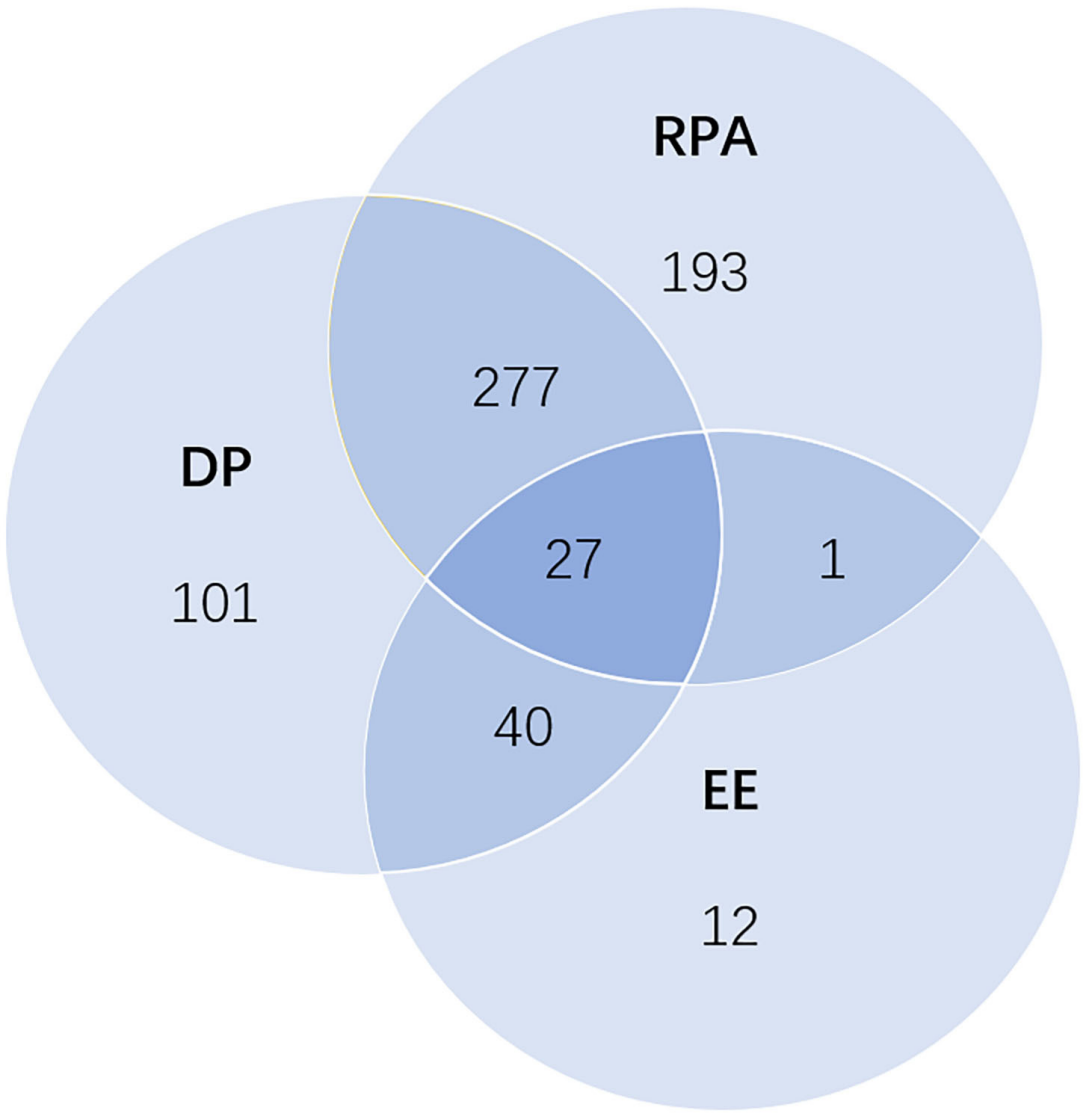
Venn diagram of the distribution of three dimensions: emotional exhaustion (EE), depersonalization (DP), and reduced personal accomplishment (RPA). The numbers for each region represent the number of health care professionals in different situations; 27 of them had all three dimensions; 318 of them had two dimensions, including 40 for EE and DP, 277 for DP and RPA, and 1 for EE and RPA. The number of health care professionals who had only EE, only DP, and only RPA were 12, 101, and 193, respectively.

### Factors Affect the Three Dimensions of Burnout

Demographic characteristics, working factors, potential risk of infection, family factor, the result of the TCQS, and distributions of each dimension of burnout in categorical items were shown in Table 1.

EE differed across working hours per week (*P* = 0.008), work intensity groups (*P* < 0.001), night shift quantity groups (*P* < 0.001), all the potential risk of infection items (*P* < 0.001), family factor (*P* < 0.001), and coping styles (*P* < 0.001). DP differed across age groups (*P* = 0.015), title groups (*P* = 0.006), night shift quantity groups (*P* = 0.005), symptom groups (*P* = 0.008), and coping styles (*P* < 0.001). About RPA, title groups (*P* = 0.013), working place groups (*P* = 0.011), and coping styles (*P* < 0.001) may be associated factors.

There was no statistical difference between the three dimensions in gender and occupational groups. Meanwhile, working place (high or low infection region) and coping style could affect the positive distribution of three dimensions simultaneously. Other factors played different roles in different burnout dimensions.

### Independent Influencing Factors of the Three Dimensions

After statistical analysis of collinearity among those factors, no significant collinear variables were found. Therefore, stepwise binary logistic regression was used to select the independent influencing factors from all column factors in Table 1. In logistic regression, the dependent variables were EE, DP, and RPA, respectively (Table 2). Firstly, higher work intensity (OR = 3.30, 95%CI: 1.86–5.58), more night shift quantity (OR = 2.08, 95%CI: 1.38–3.15), having symptoms about COVID-19 (OR = 3.29, 95%CI: 1.98–5.48), working in the high infection region (OR = 2.20, 95%CI: 1.28–3.78), and negative coping style (OR = 1.99, 95%CI: 1.21–3.26) were associated with a higher incidence of EE. Secondly, working in the high infection region (OR = 0.70, 95%CI: 0.53–0.94) was associated with a lower incidence of DP. But senior title (OR = 1.35, 95%CI: 1.00–1.82), more night shift quantity (OR = 1.32, 95%CI: 1.03–1.69), having symptoms (OR = 1.52, 95%CI: 1.07–2.17), and negative coping style (OR = 3.47, 95%CI: 2.54–4.73) were associated with higher DP. Thirdly, two items were associated with a higher incidence of RPA: senior title (OR = 1.43, 95%CI: 1.08–1.90) and negative coping style (OR = 1.82, 95%CI: 1.35–2.45). On the contrary, HCPs working in the high infection region (OR = 0.66, 95%CI: 0.51–0.87) tended to show a lower incidence of RPA.

**Table 2:** Stepwise binary logistic regression for the three dimensions (EE, DP, and RPA).

**Items**	**EE**		**DP**		**RPA**	
	**OR (95%CI)**	***P***	**OR (95%CI)**	***P***	**OR (95%CI)**	***P***
Senior title	\	\	1.35 (1.00 1.82)	0.050^*^	1.43 (1.08 1.90)	0.012^*^
Higher work intensity	3.30 (1.86 5.85)	<0.001^***^	\	\	\	\
More night shift quantity	2.08 (1.38 3.15)	0.001^**^	1.32 (1.03 1.69)	0.026^*^	\	\
Having symptoms	3.29 (1.98 5.48)	<0.001^***^	1.52 (1.07 2.17)	0.019^*^	\	\
Working in high infection region	2.20 (1.28 3.78)	0.004^**^	0.70 (0.53 0.94)	0.016^*^	0.66 (0.51 0.87)	0.003^**^
Negative coping style	1.99 (1.21 3.26)	0.007^**^	3.47 (2.54 4.73)	<0.001^***^	1.82 (1.35 2.45)	<0.001^***^

* *p < 0.05*;

** *p < 0.01*;

*** *p < 0.001*.

\, *Some factors did not come into the stepwise binary logistic regression*.

Working in varying degrees of infection regions was proved to be the independent influencing factors in all three dimensions. Working in the high infection region may be a protective factor in DP and RPA but a risk factor in EE. Some items in the four aspects were also the independent influencing factors in one or two dimensions. More night shift quantity and having symptoms about COVID-19 may play as risk factors in EE and DP. The senior title could cause an increased rate in RPA.

### The Score of Three Dimensions in Different Working Place and Coping Style Group

The scores of three different dimensions were calculated by grouping the two factors (working place and coping style) and presented as the mean plus or minus one standard deviation (Table 3). Except that the workplace could not significantly change the score of EE (*P* = 0.443), the scores of the three dimensions all showed a significant difference in groups categorized by these two factors, respectively.

**Table 3:** The score of three dimensions in different working place and coping style group.

**Items**	**EE (mean ± SD)**	**DP (mean ± SD)**	**RPA (mean ± SD)**
Working place	*P* < 0.001^***^	*P* = 0.443	*P* = 0.008^**^
High infection region	12.80 ± 0.35	5.58 ± 0.25	12.47 ± 0.39
Low infection region	12.23 ± 0.24	5.34 ± 0.19	13.85 ± 0.34
Coping style	*P* < 0.001^***^	*P* < 0.001^***^	*P* < 0.001^***^
Negative	13.49 ± 0.37	7.44 ± 0.30	15.25 ± 0.45
Positive	10.42 ± 0.24	4.53 ± 0.17	12.30 ± 0.30

* *p < 0.05*;

** *p < 0.01*;

*** *p < 0.001*.

## Discussion

### Main Findings and Previous Experience From China

This study investigated job burnout and the relationship between related factors and the three dimensions of burnout in HCPs across China against the backdrop of the epidemic of COVID-19. Chinese HCPs experienced multiple prevalent burnout symptoms in different dimensions. Higher work intensity due to the epidemic of COVID-19, senior title, more night shift quantity, having symptoms, and negative coping styles could increase the risk of job burnout. The effects of working in a high incidence area on different burnout dimensions were somewhat paradoxical.

In this study, the job burnout (which included mild, moderate, and severe burnout) rate in HCPs was 73.98%; among them, the sum of moderate and severe job burnout ratio is 39.20%. We compare these results with some studies, which also selected CMBI to assess burnout, in China in recent years during non-epidemic periods. The most recent study started in November 2018 and ended in March 2019. The study contained 514 intensive care unit physicians and nurses, 56.03% of whom reported varying degrees of burnout (33). Next, a cross-sectional study of 2,502 nurses conducted in 2017 showed that the prevalence of job burnout was 64.06%, with 30.14% being moderate and severe combined (34). In 2016, a study found that the overall prevalence of all degrees of burnout was 85.79%, and the breakdown according to severity is as follows: 40.0% mild, 27.2% moderate, and 7.4% severe burnout among 2,617 participating Chinese doctors (35). A study that took place in 2013 containing 1,435 nurses from two large general hospitals showed that the overall prevalence of all degrees of burnout was 74.6%, with 40.0% mild, 27.2% moderate, and 7.4% severe burnout (17). From December 2009 to February 2011, a questionnaire survey across China showed that of 2,530 physicians, 34.2% were experiencing moderate burnout and 5.5% were experiencing severe burnout (36). Taking all the above studies together, we concluded that the proportion of Chinese medical workers suffering from moderate to severe burnout did increase during the epidemic. It is worth noting that previous studies had shown a long-term psychological and occupational effects of SARS (severe acute respiratory syndrome coronavirus) that it could even significantly increase the level of burnout among health care workers who cared for SARS patients compared to those who did not after 13 to 26 months (37). Perhaps COVID-19, which is similar to SARS, will have a similar effect, which needs further study.

Anyway, in the context of a viral pandemic, many clinicians had a heavy medical burden; the high rate of job burnout in China, which was associated with poor HCPs' health and decreased quality of medical care, was not optimistic (38, 39).

### Other Studies Addressing Burnout During the COVID-19 Pandemic

As of November 2020, the epidemic is still widespread. Several studies on burnout among HCPs in COVID-19 have been published. These studies also revealed that burnout was common among HCPs in many different countries. A cross-sectional study about burnout between normal ward workers and frontline workers in COVID wards in Romania showed that 76% of the sample reported burnout (40). Another cross-sectional study in Northern Italy also revealed that 76% of health professionals working in an institution had been burned out (41). A large sample survey in the USA found that HCPs who contracted COVID-19 reported higher levels of burnout (42). All of these studies used the Maslach Burnout Inventory. Therefore, the situation of burnout among health care workers around the world is serious and is of concern.

### Risk Factors of Burnout

Risk factors for job burnout have been widely studied. It is agreed that the level of job burnout varies from different occupations and different countries (5, 43). Younger is widely believed to have an association with higher burnout levels since new employees have less experience in dealing with problems at work (5). This study was a cross-sectional study, but there was an underlying chronological relationship between internal factors (except coping style) and burnout. It showed that age had an impact on the level of DP in HCPs according to univariate analysis. Among a great number of studies, there are a few to compare the differences between doctors and nurses on job burnout levels. We performed this comparison and found no statistical difference in three dimensions of burnout between these two occupations. The same result was observed in the factor of gender.

Working factors, assessed by three factors in this study: long working hours, high work intensity, and more night shift, contributed to the occurrence of job burnout together, especially in the EE dimension. Stepwise logistic regression confirmed the impact of these work-related stressors. The high workload can drain employees' mental and physical strength and may cause exhaustion through the process of health impairment (6, 44). Based on the classical job demands–resources model of job burnout, one solution to this dilemma is to provide more job resources for HCPs, such as social support, potential promotion, and learning opportunities (45, 46). HCPs who were isolated from family members may not be able to undertake the obligation of taking care of the elderly and children as well as daily housework. This kind of work–family conflict had been proved to harm job burnout (47), which had also been detected in our study. On the other hand, isolation from the family caused a lack of emotional support that also could elicit burnout (48, 49).

Due to the high infectivity of COVID-19, HCPs who were directly contacted with patients had a higher level of EE, and those who had suspicious symptoms were more susceptible to EE and DP. However, the influences of working place on three dimensions of job burnout were not consistent. HCPs working in the high infection region have a higher level of EE but a lower level of DP and RPA than those working in the low infection region. Such results seemed to be contradictory but may have a possible explanation. Short-term exposure to this epidemic may inspire the dedication of medical individuals and compassion for patients, as well as a sense of pride, leading to the decline of DP and RPA symptoms. However, we were not able to figure out the long-term effect of this exposure on job burnout due to the limitations of the cross-sectional study.

### Coping Style and Burnout

Our research has confirmed a strong link between job burnout and coping styles, and negative coping can increase the score and the corresponding positive incidence in three dimensions. This result was consistent with many studies (17, 18). The process of medical behavior is full of challenges, requiring HCPs to solve one problem after another. If the problem is not solved successfully, HCPs might have negative emotions (e.g., sadness, despondency, irritation, or hopelessness) (50). When these negative emotions are repeated among HCPs, they gradually become emotionally exhausted. Thus, the HCPs who tend to choose positive coping strategies are less likely to develop burnout. Of course, the mechanism for the link between coping style and burnout should be complex. The positive coping style of problem-focused coping was even linked to improved psychological health (such as depression and anxiety) (51). There was a study focused on cognitive-behavioral and psychoeducational intervention on coping strategies, and it showed that the higher use of active coping strategies resulted in a decrease in levels of burnout (52). In such an emergent bio-disaster, guiding HCPs to adjust on their coping styles in positive and rational ways may help them reduce the occurrence of job burnout.

### Three Dimensions of Job Burnout

As mentioned above, the positive rates of the three dimensions of burnout (emotional exhaustion, depersonalization, and reduced personal accomplishment) were 9.09, 50.57, and 56.59%, respectively, in our study. It was very interesting but difficult to explain the phenomenon that there was a large difference in the positive rate between the EE dimension and the remaining two. According to Maslach et al., there is a hypothesis about these three dimensions: it is a different sequential progression over time; the occurrence of one dimension precipitates the development of another. According to this model, emotional exhaustion occurs first, leading to the development of depersonalization, which leads subsequently to reduced personal accomplishment (5). So, the high level of DP and RPA might indicate that burnout among HCPs has been developing over time. The lower proportion of EE may be due to the impact of this outbreak. During the outbreak, the Chinese people and the government paid more attention to doctors, and many heart-warming events about HCPs happened, like the fact that many insurance companies offered free coverage to some HCPs. To some extent, the pride and emotional needs of HCPs were greatly satisfied.

### Limitations

The study also had the following limitations. This study was cross-sectional in design. Coping styles and job burnout were measured simultaneously. Therefore, it was impossible to draw a causal relationship between them. Selection bias cannot be avoided. More risk factors and their mechanisms should be investigated in further studies. The study did not include any infected health care workers, but their job burnout and psychological status were also highly worth studying.

## Conclusion

There was a certain degree of job burnout among Chinese HCPs during the COVID-19 outbreak. The rate of moderate and severe burnout had increased compared to non-epidemic periods. And some of the factors (higher work intensity, more night shift quantity, having symptoms, working in the high infection region) associated with this outbreak had been shown in our study to be closely related to burnout. We should continue to pay attention to the status of job burnout among HCPs and take preventive interventions in advance. Public health interventions should be taken to reduce the working intensity, the number of night shifts, and the risk of infection of HCPs; psychological interventions should also be taken to help more HCPs adopt the positive coping style, which would help to reduce job burnout.

## Data Availability Statement

The original contributions presented in the study are included in the article/Supplementary Material, further inquiries can be directed to the corresponding author/s.

## Ethics Statement

The studies involving human participants were reviewed and approved by the Ethics Committee of Union Hospital, Tongji Medical College, Wuhan China. Written informed consent for participation was not required for this study in accordance with the national legislation and the institutional requirements.

## Author Contributions

XLiu, JC, and TB were responsible for draft writing, conceived the idea for the article, analyzed the data of this study by software, and wrote the final manuscript. DW, XLi, EW, YJ, YM, and CY contributed to data collection in the fieldwork and gave suggestions for data analysis. CLu, LZ, CLi, and YZ assisted in entering raw data into statistical software. LY and JS contributed to check the analyses, and critically reviewed the manuscript. TB and XH contributed to design the study, supervise, and make the final decision to contribute to the article. All authors contributed to and approved the final report.

## Conflict of Interest

CLi and YZ were employed by the company Wuhan Estrip Tech Co., Ltd. The remaining authors declare that the research was conducted in the absence of any commercial or financial relationships that could be construed as a potential conflict of interest.
